# Imaging of developmental delay in black African children: A hospital-based study in Yaoundé-Cameroon

**DOI:** 10.4314/ahs.v23i1.73

**Published:** 2023-03

**Authors:** Seraphin Nguefack, Nasser Ndongafack Fongue, Daniel Armand Kago Tague, Ulrich Igor Mbessoh Kengne, Jean Roger Mouliom Tapouh, Félicitée Nguefack, Andreas Chiabi, Boniface Moifo

**Affiliations:** 1 University of Yaounde I Faculty of Medicine and Biomedical Sciences, Pediatrics; Yaounde Gynaeco-Obstetric and pediatric Hospital, pediatrics; 2 University of Yaounde I Faculty of Medicine and Biomedical Sciences; 3 University of Dschang; 4 University of Yaounde I Faculty of Medicine and Biomedical Sciences, Radiology; Yaounde Gynaeco-Obstetric and pediatric Hospital, radiology

**Keywords:** developmental delay, brain MRI, CT scan, children

## Abstract

**Background:**

The purpose of this study was to describe the anomalies observed on imaging for developmental delay in black African children.

**Methods:**

It was a descriptive cross-sectional study, which included children aged between 1 month to 6 years with developmental delay and had done a brain MRI and/or CT scan.

**Results:**

We included 94 children, 60.6% of whom were males. The mean age was 32.5 ± 6.8 months. A history of perinatal asphyxia found in 55.3% of cases. According to the Denver developmental II scale, profound developmental delay observed in 35.1% of cases, and severe developmental delay in 25.5%. DD was isolated in 2.1% of cases and associated with cerebral palsy, pyramidal syndrome, and microcephaly in respectively 83%, 79.8%, and 46.8% of cases. Brain CT scan and MRI accounted for 85.1% and 14.9% respectively. The tests were abnormal in 78.7% of the cases, and cerebral atrophy was the preponderant anomaly (cortical atrophy = 80%, subcortical atrophy = 69.3%). Epileptic patients were 4 times more likely to have abnormal brain imaging (OR = 4.12 and p = 0.05),. We did not find a link between the severity of psychomotor delay and the presence of significant anomalies in imaging.

**Conclusion:**

In our context, there is a high prevalence of organic anomalies in the imaging of psychomotor delay, which were dominated by cerebral atrophy secondary to hypoxic ischemic events.

## Introduction

Psychomotor delay is a retardation in the development of the child's faculties with respect to language, motor skills, and social development, compared to the norms defined for its age 1–3. Although according to recent epidemiological data it constitutes 1 to 3% of global morbidity, there is still a geographical variability of the morbid burden attributable to it 4. In countries with low economic resources like Cameroon, this prevalence appears to be higher, and estimated at 7% according to a hospital-based study conducted by Nguefack et al in 2013 1. This is to say that developmental delay is not scarce, and also represents a major health issue. The resort to neuroradiological examinations (MRI and CT) is of fundamental necessity in the etiologic investigation approach for children's psychomotor regression 5,6. The identification of this aetiology makes possible to optimize the management of these patients and also, identifying the underlying organic anomaly is of a prognostic interest 3,7,8. Additionally, because it enables the establishment of an organic cause of psychomotor delay and precedes the realization of more specific tests such as molecular biology, when necessary, recent works recommend brain MRI as gold standard^3^,^5^,^6^,^9^–^11^. However, CT scan is an acceptable alternative in the absence of MRI and for the search of cerebral calcifications^5^,^6^,^8^,^9^.

In Cameroon, several studies have already been carried out on developmental delay, but none has been devoted to etiological research. Our context characterized by the availability of CT scans, the recent introduction of MRI, and the virtual inaccessibility of molecular biology and genetics techniques. In this context, we undertook this study to describe the anomalies observed on imaging of developmental delay in Yaounde Gynaeco-Obstetric and Pediatric Hospital.

## Methods

### Study setting and study design

We conducted a descriptive cross-sectional study over a period of 09 months, from 1st September 2017 to 30 June 2018, in the pediatric neurology unit of the Yaounde Gynaeco-Obstetric and Pediatric Hospital during the follow up in the outpatient clinic.

### Sample

Sampling was consecutive and non-exhaustive. The study consecutively included all children aged 1 month to 6 years with a developmental delay based on clinical examination, who had performed a brain MRI and/or CT scan. Patients whose psychomotor regression was secondary to head trauma and those whose guardians had not consented to their participation in the study were excluded.

### IRB Approval

The Institutional Ethics and Research Committee of the Yaoundé Gynaeco-Obstetric and Pediatric Hospital and Faculty of Medicine and Biomedical Sciences of the University of Yaounde I approved the study.

### Data collection method and tool

After obtaining the informed consent of the guardians, the data were collected by interviewing the guardians and a complete neurological and general clinical examination was performed. A consensual interpretation of the brain MRI and CT scans of the selected patients was performed by a neuro-pediatrician and a pediatric radiologist. The variables studied were sociodemographic, past history, clinical and radiological. Regarding clinical variables, microcephaly was considered as a head circumference (HC) <-2 Z-score on WHO growth curves. Severe acute malnutrition was defined as a weight-for-height ratio (W/H) <-3 Z-score on WHO growth charts. Growth retardation was defined by a length/height for age ratio (L/A) <-2 Z-score on WHO curves. The evaluation of the level of psychomotor development was carried out using the Denver II scale, which is a scale that evaluates the level of psychomotor development in 04 items: gross motor skills, fine motor skills, language and social contact^12^. The development quotient (QD) was calculated from the formula: Developmental Quotient = (Developmental Age/Real Age) x 100. The psychomotor delay was classified according to the WHO in mild (50≤QD≤69), moderate (35≤QD≤49), severe (20≤QD≤34) and profound (QD<20).

### Statistical analysis

Data was analyzed using SPSS software version 23.0. The Chi-2 test was performed to make comparisons between categorical variables. A p-value <5% was considered as statistically significant.

## Results

We included 94 children aged under 6 years, 57 of whom were boys (60.6%), a sex ratio of 1.54. The mean age was 32.5 ± 6.8 months and patients aged 2 to 6 years accounted for 61.7% of the sample. The main past history found were perinatal asphyxia in 52 cases (55.3%), congenital microcephaly in 6 cases (6.4%), and 5 cases of viral infection (CMV) in pregnancy (5.3%). Evaluation of development with the Denver II scale found 33 children (35.1%) with profound development delay and 25.5% were severe.

Concerning clinical features, we found that 78 children had cerebral palsy (83%), 75 (79.8%) a pyramidal syndrome, 44 (46.8%) a microcephaly, 32 (34.0%) an epilepsy, 28 (29.8%) a growth retardation, 7 (7.4%) a severe acute malnutrition, 6 (6.4%) a choreoathetosis, and 5 (5.3%) had a facial dysmorphism.

**Figure 1:**
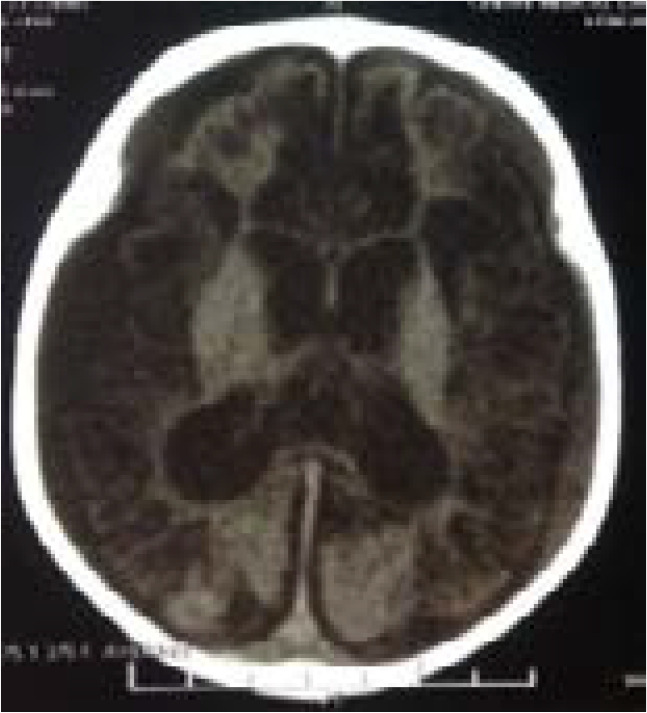
diffuse cortico-cortical atrophy associated with multiple porencephalic cavities and cerebral softening

**Figure 2:**
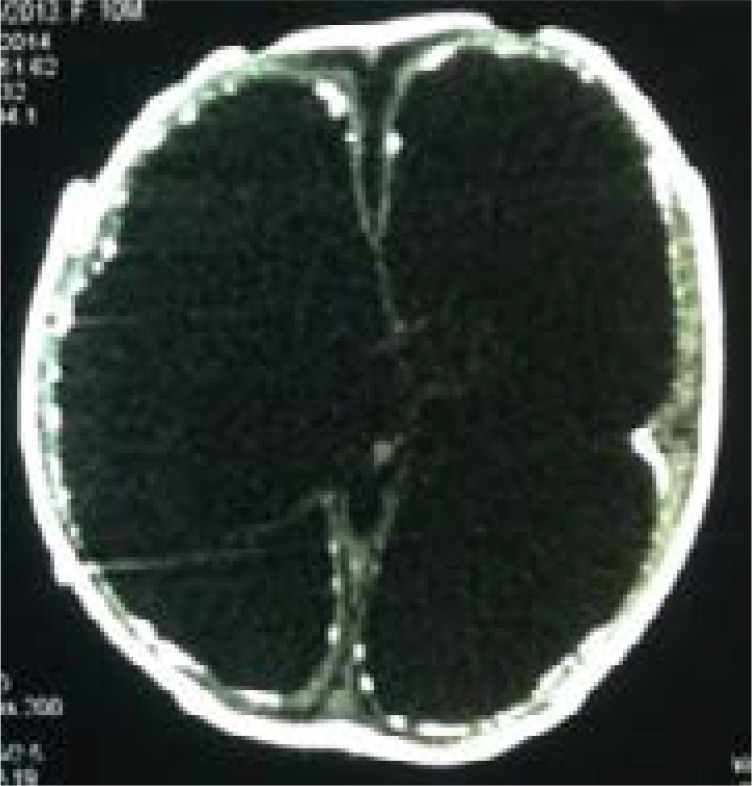
hydrocephalus, with sequential subependymal chain calcifications of cytomegalovirus embryofetopathy

**Figure 3:**
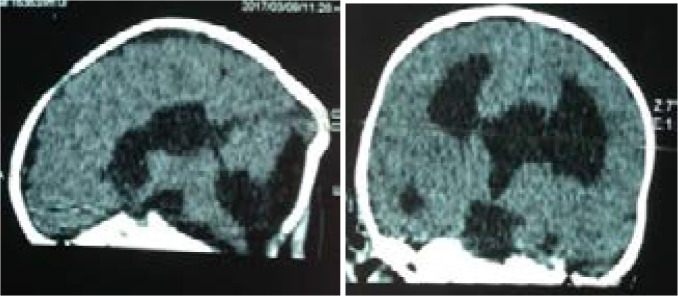
Brain CT without injection in sagittal and axial parenchymal window: left cortical and subcortical parafalcoreal hypodensity, associated with colpocephaly, complete agenesis of the corpus callosum and agenesis of vermis

**Figure 4:**
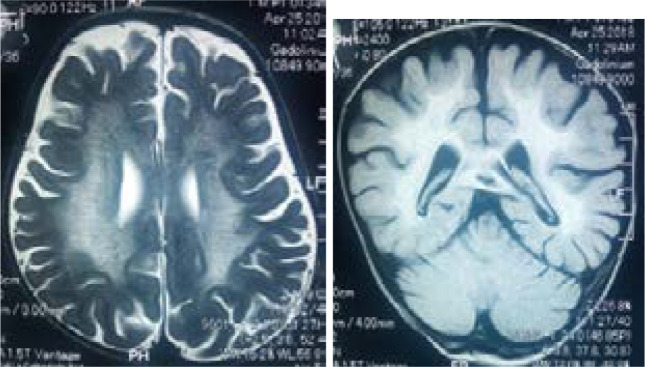
Brain MRI without contrast injection in axial section T1, T2 and FLAIR sequence: T2 and FLAIR hyperintensity of the deep white matter bilaterally and symmetrically and T1 hyposignal associated with corpus callosum involvement compatible with adrenoleukodystrophy

The 94 imaging examinations consisted of 85.1% (n= 80) brain CT scans and 14.9% (n= 14) brain MRIs. The prevalence of anomalies on imaging was 78.7%. The main etiologies observed were cortical atrophy (80%) and subcortical atrophy (69.3%) secondary to a hypoxic ischemic event (Figure 1), congenital CMV infection (10.7%) (Figure 2), corpus callosum agenesis (4%) (Figure 3), and adrenoleukodystrophy (4%) (Figure 4). Table 1 shows the distribution of patients according to anomalies observed in cerebral imaging.

**Table 1:** Distribution of patients according to the etiologies observed in cerebral imaging

Etiologies	Number (%)
**Hypoxic ischemic sequalae**	
Cortical Atrophy	60 (80.0)
Sub cortical Atrophy	52 (69.3)
Cortical and sub cortical Atrophy	49 (65.3)
Cortical and sub cortical atrophy with area of porencephaly	18 (24.0)
Cortical and sub cortical Atrophy with Watershed hypodensity	13 (17.3)
**Cerebral malformation**	
Agenesis of the corpus callosum	3 (4.0)
Vermian agenesis	3 (4.0)
**Congenital CMV infection**	8 (10.7)
**Metabolic disease**	
Adrenoleukodystrophy	3 (4.0)
**Hydrocephalus**	4 (5.3)
**Neurodegenerative disease**	3 (4.0)

Epileptic patients were 4 times more likely to have abnormal brain imaging (OR= 4.12 and p= 0.05), whereas those with chorea-athetosis were 20 times more likely to have normal brain imaging (OR= 0.05 and p= 0.01). We did not find any link between the severity of psychomotor delay and the presence of significant abnormalities in imaging (Table 2).

**Table 2:** Association between clinical feature, degree of developmental delay and abnormal imaging

	Abnormal imaging		
Variables	Yes n (%)	No n (%)	OR (IC 95%)
**Clinical abnormalities**			
Epilepsy	29 (90.6)	3 (9.4)	**4.12 (0.98–17.38)** *
Microcephaly	0 (0.0)	2 (100)	2.44 (0.76–7.85)
Choreoathetosis	1 (16.7)	5 (83.3)	**0.05 (0.01–0.53)** **
**Degree of developmental delay**			
Mild	14 (73.7)	5 (26.3)	0.7 (0.22–2.48)
Moderate	16 (88.9)	2 (11.1)	2.48 (0.57–17.17)
Severe	18 (75)	6 (25)	0.75 (0.25–2.41)
Profound	26 (78.8)	7 (21.2)	1.01 (0.36–2.99)

* p= 0.05

** p= 0.01

## Discussion

In this study on the imaging of developmental delay in children, we report data on the clinical profile and the etiologies observed in the brain MRIs and CT scans of 94 children followed up at the pediatric neurology unit of the Yaounde Gynaeco-obstetric and Pediatric Hospital.

Our study reports an average age of 32.5 ± 6.8 months, with a male predominance (60.6%). It is indeed at this age, as noted by Nguefack and Amadou, that most parents notice and get upset about delays in psychomotor acquisitions of their offspring 1,13. Estrogen appears to play a protective role in the female fetal brain against ischemia and anoxia 14, which explains the preponderance of male cases that we observe in most studies 15.

In Cameroon, perinatal asphyxia is also positioned as the main risk factor for psychomotor delays in children 1. The irreversible lesions that hypoxic ischemic events cause on the cells of the central nervous system are also responsible for the clinical manifestations of cerebral palsy (83%) and epilepsy (34%), which the literature corroborates^1^,^16^,^17^.. Van Karnebeek et al reported a prevalence of 18%^18^, while it was 46.8% in our study. This difference can be accounted for by the frequency of perinatal asphyxia in our environment, responsible for cerebral atrophy of hypoxic ischemic origin by destruction of neuronal and glial fibers, which in turn induces precocious fusion of skull sutures. As for the growth retardation in children with developmental delay, a contrast is clearly visible between the 29.8% reported by our study and the 2% of the Aloui *et al* 9. The high prevalence of cerebral palsy in our environment could explain this difference in frequency. Nguefack et al reported that underweight and growth retardation were the most common forms of malnutrition in children with cerebral palsy 15. It is also well established that the poor growth and weight of children with cerebral palsy have multifactorial etiologies, associating nutritional factors related to poor nutrient intake (pharyngeal motor disorders), environmental factors, and endocrine factors including growth hormone deficiency 19. Denver developmental screening test II (DDSTII) is one of the most widely used scales in the developmental assessment of children with neurological disorders 1,12,14. However, it is clear that many studies that have focused on the theme of psychomotor delay of the child didn't mention the distribution of patients according to the severity of the condition. Therefore, the proportions of 35.1% and 25.5% for profound and severe developmental delay respectively were prominent in our sample.

The unavailability and financial accessibility of the MRI still remain a challenge in many countries of sub-Saharan Africa, which favors the use of CT scan 13. This explains why of the 94 imaging examinations performed, there were 80 (85.1%) brain CT scans and 14 (14.9%) brain MRI scans. In 78.7% of cases, imaging was abnormal. This is a highly frequent finding that is not unique to our context because many studies conducted in Asia, Europe, India, and America all report a prevalence above 50%^4^,^6^,^9^,^10^,^13^,^20^.

Similarly, concerning the etiologies observed, cerebral atrophy (cortical and subcortical) and cerebral malformations were found to be the majority in our study as well as in the literature 4,6,7,13,17.

On the other hand, due to a small sample size of choreoathetosis, (), this clinical sign is rather associated with the absence of anomalies in brain imaging. There are abnormalities associated with bilirubin encephalopathy on MRI in the globus palladus 20. Finally, like Kjos et al 21, we did not find any link between the severity of psychomotor retardation and the presence of abnormalities on imaging that was possible because that anomalies might have been missed as most participants only had CT scans done which might miss abnormalities.

## Conclusion

In our context, we have a high prevalence of organic anomalies in imaging, which are dominated by cerebral atrophy due to hypoxic ischemic events. These findings support the systematic realization of a brain MRI or alternatively a brain CT scan for every child presenting with psychomotor retardation.
